# Addressing healthy aging populations in developing countries: unlocking the opportunity of eHealth and mHealth

**DOI:** 10.1186/s12982-014-0021-4

**Published:** 2014-12-31

**Authors:** Cesar Henriquez-Camacho, Juan Losa, J Jaime Miranda, Natalie E Cheyne

**Affiliations:** Instituto de Medicina Tropical Alexander von Humboldt, Lima, Peru; Infectious Diseases/Internal Medicine Unit. Hospital Universitario Fundacion Alcorcon, Madrid, Spain; CRONICAS Center of Excellence in Chronic Diseases, Universidad Peruana Cayetano Heredia, Lima, Peru; School of Medicine, Universidad Peruana Cayetano Heredia, Lima, Peru; School of Medicine, University of Manchester, Manchester, UK

**Keywords:** eHealth, Ageing population, Technology, Developing world, mHealth

## Abstract

Aging societies worldwide propose a significant challenge to the model and organisation of the delivery of healthcare services. In developing countries, communicable and non-communicable diseases are affecting how the ageing population access healthcare; this could be due to varying reasons such as geographical barriers, limited financial support and poor literacy. New information and communication technology, such as eHealth have the potential to improve access to healthcare, information exchange and improving public and personalised medicine for elderly groups. In this article we will first frame the context of information and communication technologies in light of an aging landscape. We will also discuss the problems related to implementing the needed infrastructure for uptake of new technology, with particular emphasis on developing countries. In so doing, we highlight areas where newer technologies can serve as promising tools or vehicles to address health and healthcare-related gaps and needs of elderly people living in resource-constrained settings.

## Introduction

**Figure 1:**
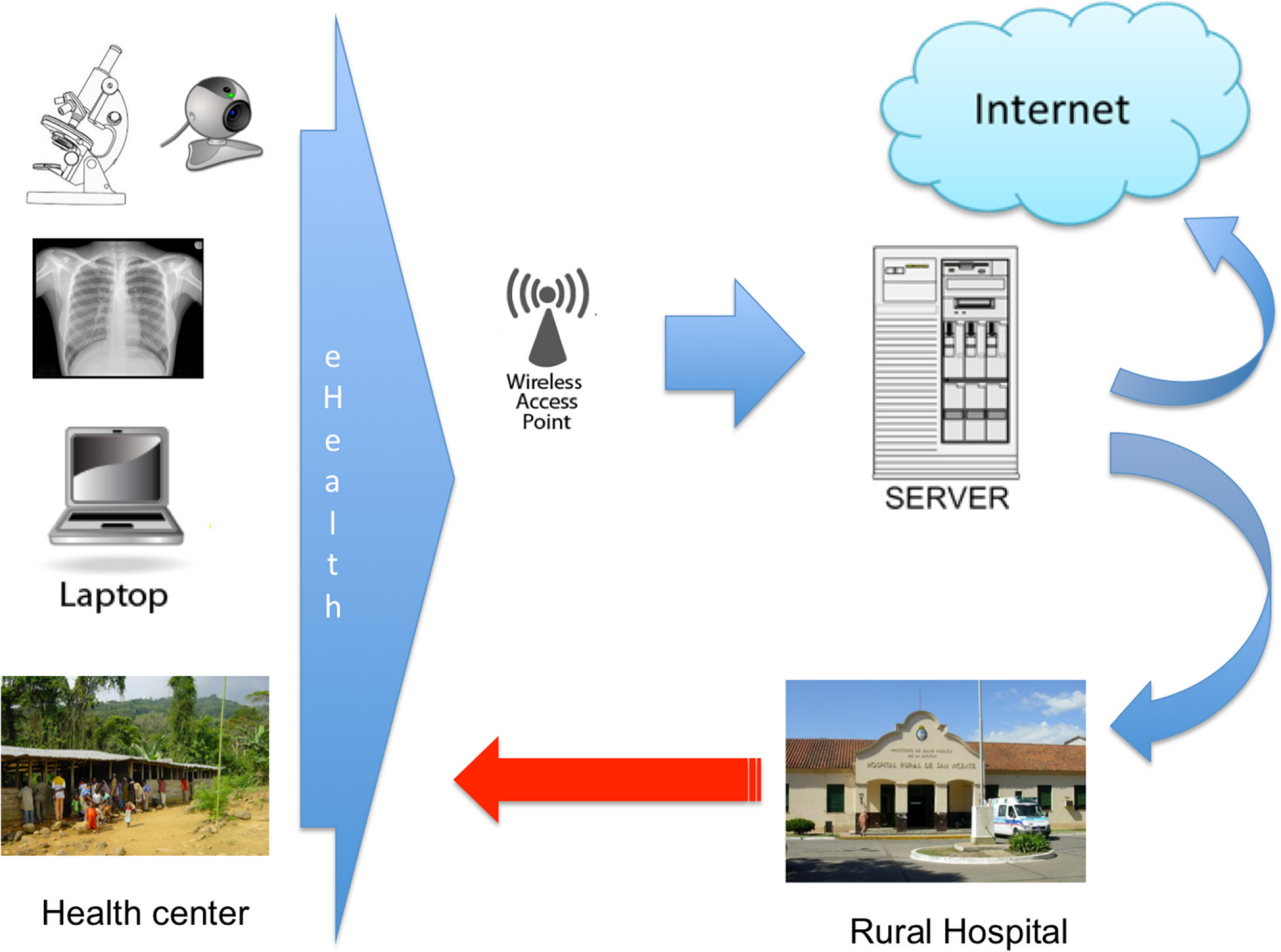
**Architecture of eHealth.** Patients in rural areas visit medical centers. Basic medical data (radiology, laboratory/urinary test, microbiology, photos/videos, etc.) is sent to ehealth platform (network server) at secondary referral hospital where specialists analyze information. Finally, diagnosis and prescription information is sent to healthcare workers.

Information and communication technology (ICT) stresses the role of unified communications and the integration of telecommunications, electronic devices, and audio-visual systems, all of which enables users to access, process, store, and transmit information. ICT has become indispensable in different sectors of our society, including healthcare. eHealth is a relatively recent term for healthcare practice supported by ICT, dating back to at least 1999 (see List of Areas of eHealth for elderly) [1,2]. eHealth technologies have the potential to improve access to healthcare as well as aiding information exchange, reducing costs and improving public and personalized medicine [3-5] (See Figure 1).

**Areas of eHealth for elderly population**

- Electronic health records
- e-Prescribing
- Telemedicine
- Consumer health informatics
- Health knowledge management
- mHealth
- Healthcare information systems
- Social media

In parallel, mobile networks and portable devices demonstrate an enormous presence and increasing penetration into the global market. Mobile phone messaging has been used to provide appointment reminders, compliance with medications- to monitor chronic conditions and to provide psychological support not only in developed countries but in developing settings [6,7]. Mobile phones are increasingly accessible worldwide and have a growing influence on society. There were an estimated 6.8 billion mobile-phone subscriptions being used across the world in 2013 (world population: 7.1 billion people) [8].

As expected, it is the younger population who can relate more with the use of ICT when compared with the older generation of whom tend to be more vulnerable to be disease and are afflicted with morbidity and disability, which may limit learning new ways of doing things [9,10].

## Problems related to age and technology

### Are elderly people catching up with new technologies?

One important problem for elderly is the lack of familiarity and mastery of ICT skills, and more so with advancing age [11].

In parallel, world’s population is ageing as a result of both longer life expectancy and declining fertility rates. From the year 2000 to 2050, the percentage of the world’s population of over 60 years of age, is expected to double from 11% to 22% [12].

A European survey conducted in seven countries revealed that among the elderly there was an increase in Internet health users 22.6% in men and 9.8% in women [13]. Some factors that can influence the acceptance or use of technology include: concerns regarding technology and high costs, expected benefits, need for technology, and social influence [14,15].

Older adults who have health problems and feel socially isolated have expressed anxiety or a lack of interest when it comes to using electronic devices or the Internet [16]. Despite their rapidly increasing rate of Internet use, older adults still lag behind younger adults when using new technologies in their everyday life which was put down to declines in cognition, vision, hearing and motor skills [17]. The Pew Internet Research Centre survey conducted on 2013 found that only 56% of Americans over age 65 in the United States are connected to and use the Internet, compared with 83% of those in the 50 to 64 age group [18]. Although, older adults who have had the chance to learn how to use new technologies have been shown to experience a number of positive outcomes compared to those who are not online; including a higher sense of empowerment, self-efficacy and wellbeing. (See List of Benefits of consumer online health-information seeking) [19-22].

**Benefits of consumer online health-information seeking** [21]

- Widespread access to health information in remote areas and vulnerable populations.
- Equity in access to health information.
- Interactivity with the web contents, in the web 2.0 entorno and social media. Interactivity facilitates interpersonal interaction.
- Tailoring information: interactive health communication offers the potential for more personalized service and users can select sites, links and specific messages based on knowledge, educational or language level, need, and preferences.

Some studies have found that older adults with a higher socioeconomic status (based on education, health literacy, and income) were more likely to use the Internet [23-25]. A study published by Choi et al. showed that having a chronic medical condition increased the use of the internet for health-related tasks. However, older adults who used the Internet for only email/texting purposes were the most socially and economically disadvantaged group of Internet users [26].

### mHealth and aging

**Figure 2:**
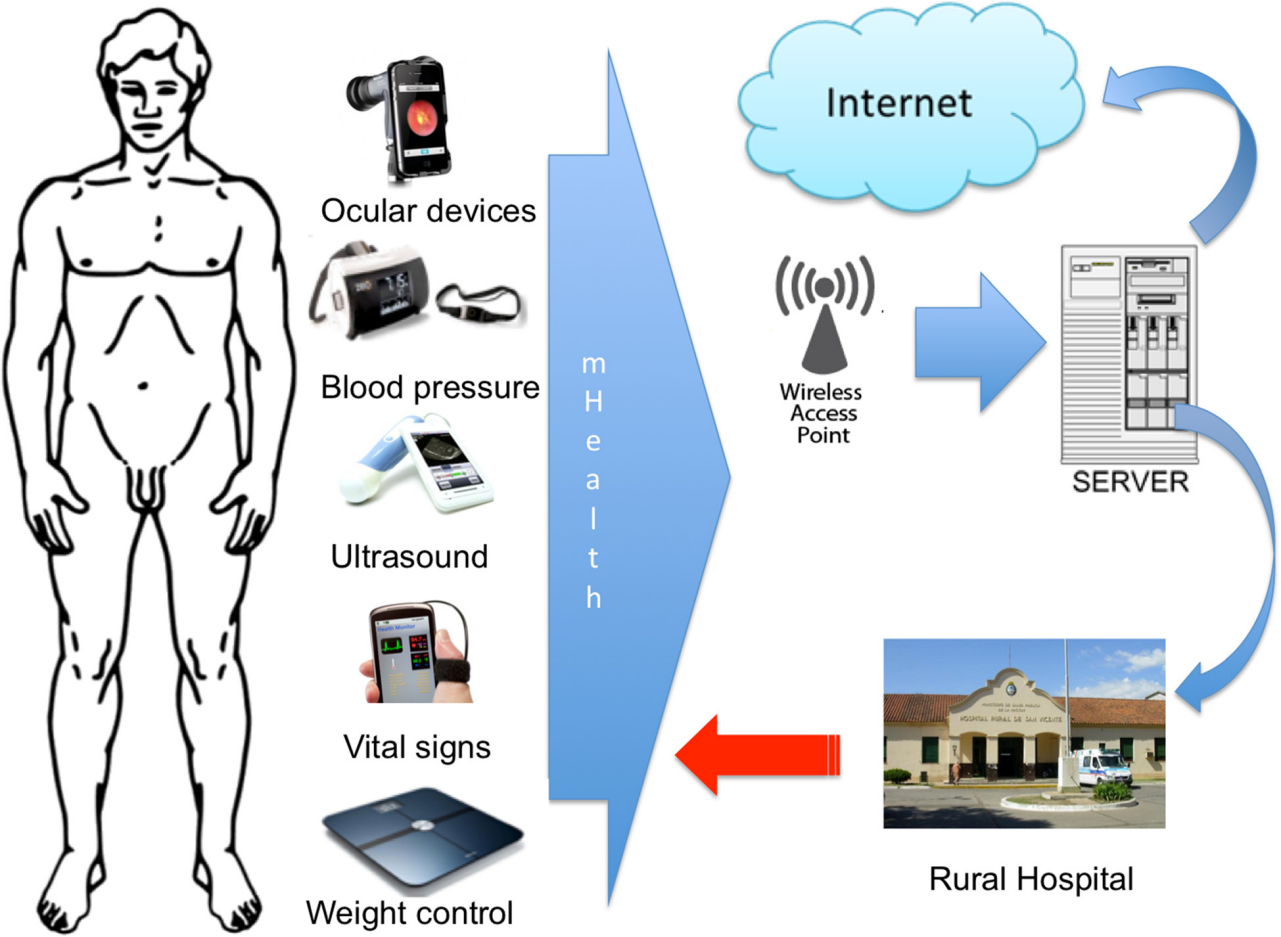
**Architecture of mHealth.** Smartphone’s patient with different devices/sensors (accelerometer, pulse and oxygen blood sensor, blood pressure, body temperature, weight, glucometer, electrocardiogram, ocular/ultrasound devices) send information to a network server/cloud. This information can be used to monitor in real time the state of the patient. Data is visualized in real time and healthcare workers can support patients directly or share information to specialists at secondary referral hospital, if needed. Finally, diagnosis and prescription information is sent to the patient.

Mobile health (mHealth) is the practice for health-related purposes using technology on mobile devices. Robert Istepanian coined the term mHealth in 2005. Today mobile health information technologies are being used around the world to address a spectrum of disease surveillance, from health systems, health education, and communication between healthcare workers, emergency and disaster response and adherence to medication [27] (See Figure 2).

mHealth tools have been tested in low and middle-income countries, where mobile devices have the added advantage of extended battery life, simple interface, voice operability, connectivity and relative affordability [28]. Most randomized trials of mHealth interventions have employed text message reminder systems. Two systematic reviews have described the use of text message reminders to improve attendance at health care appointments [29,30]. A recent meta-analysis based on low to moderate quality evidence showed that mobile phone text messaging reminders increase attendance at healthcare appointments compared to no reminders, or postal reminders [7].

The use of mobile devices and wireless routers to gather and access health data has grown in recent years. Popular mHealth apps are used for health purposes such as counting calories, gauging nutrition and nutritional status, tracking workouts, calculating body mass index or even quitting smoking [31]. Also, mobile devices offer attractive low-cost, real-time ways to assess disease, movement, images, behaviour, social interactions, or environmental agents. As costs decrease, there is interest on the potential use of mobile communication in remote and less resourced settings in developing countries [32].

### Problems related to mHealth and aging

Many older adults have physical limitations and cognitive impairment. Motor skill and vision impairment may cause them frustration and anxiety in simple everyday tasks, not just when it comes to new technologies [33]. Poor health literacy is common in elderly patients. The lack of adequate literacy is twice as common for older and vulnerable people, [34-36] but training older adults to use the internet has showed lower anxiety, higher confidence, and higher self-efficacy towards the use of health information searches on the Internet [37].

A large survey demonstrated that people of 75 years of age or older with a higher education level had higher rates of Internet use for health reasons and a half of those with chronic diseases indicated that their use of the Internet improved not only their understanding of their chronic disease but their understanding of its treatment options [38].

The Internet is an important source of information for patients affected with chronic diseases. Chronic, non communicable diseases (NCD) are a leading cause of morbidity and mortality in this group of patients. Available data demonstrates that nearly 80% of NCD deaths occur in low- and middle-income countries [39]. All are projected to increase in the developing world as incomes rise [40,41].

One of the problems of mHealth is the small number of studies with few participants in chronic diseases in developing countries. The actual evidence is based in low to moderate quality of studies. Finally, mHealth is affected by lack of integration in other systems to exchange data in common formats, or the use of a common protocol (interoperability) [42,43].

## Developing countries and older people

Developing countries have a high incidence of communicable disease and increasing incidence of chronic NCD such as hypertension, obesity, heart disease, and diabetes. The combined effect of chronic communicable diseases or non-communicable diseases is described as a “dual burden” for developing countries [44]. Elderly people in developing countries have limited resources to access to medical care. In poor rural areas, the hospital resources are concentrated in urban areas. There are not enough health care workers in rural areas and such workers are difficult to recruit and retain. Most of the school medicines are concentrated in urban areas and new physicians prefer to practice on urban areas [45]. Another aspect related to rural areas is the lack of literacy that decreases access to electronic and learning resources and similarly, large geographical areas and distant villages are a challenge to provide access to health care in developing countries.

## Potential solutions

The potential solutions in developing countries focus mainly on making both the patient and healthcare professionals alike, aware of the new technologies available and how they can be applied routinely into the lives of patients with chronic diseases. The new technologies are tools with the objective of demonstrating how to look after patient’s lives via the tools’ integrations into daily work which should be progressive and adapted to suit each individual’s needs. We will later address in this paper the different realms in which we can develop the new technologies (See Table 1).

**Table 1:** **Problems and solutions**

**Tools**	**Problems**	**Solutions**
**Electronic mailing listing**	**Misinformation or misunderstanding**	**Clear and simple information with image or audio support. Maybe bidirectional information (provider and users) be more effective to confirm delivery messages.**
**Social Networking**	**Misunderstanding o lack of information. Loss of confidentiality.**	**Reforcement of security through education and control of accessibility.**
**Web-learning**	**1. Needs fast computers with sound/video cards and Internet connection.**	**1. Private and public founding to cover costs.**
	**2. Need logistic assistance (webmaster, programmer, editor, designer.**	**2. Free-wifi network.**
		**3. Learning skills about computers and internet navigation.**
**Telemedicine**	**1. Costs of telecommunication**	**1. Public and private support to cover costs.**
	**2. Data management of equipment**	**2. Free wifi networking for remote regions**
	**3. Technical training**	**3. Learning skills about computers and internet navigation.**
	**4. Informatic support**	
**mHealth**	**1. High costs of mobile devices**	**1. Lower costs of mobile devices. Android has a clear advantage over iOS devices in terms of price and technology.**
	**2. Security problems**	**2. Education and training in security systems to share medical information via apps.**
	**3. Internet conection**	**3. Public and private support to expand mobile and wireless network to the most remote and resource-poor environments.**

### Web surfing

The Internet has helped medical information to become widely available. Accessing information through surfing the web allows patients to learn more about diseases and medical terms that sometimes are complicated to understand. Patients and providers can use the Internet to obtain information about clinical practice guidelines, alternative treatments, and the best places for treatment of uncommon conditions; even in physically remote areas. However, the lack of control over the quality of information can result in the spread of inaccurate information, which can be an obstacle for the relationship between health provider and patients. Furthermore, it is important to note that we must also educate the population in how to use the Internet for such matters, not just create the potential to use the Internet for advancing mHealth. The web-pages themselves must be adapted to the user (appropriate language, easy to use format, easy to navigate etc.). An example of this is the webpages associate with the National Health Service (NHS) in Great Britain www.nhs.uk and National Institute of Health England www.health.nih.gov.

### Social networking

Social networking through the means of social media can be effective in health promotion; such as in encouraging healthy behaviours through carefully crafted messages in social groups. Web 2.0 has opened the access of information and interaction between people with both the same or different ideas. In rural areas with Internet access, such networks may be an efficient channel available for public information campaigns or prevention messages about smoking cessation, improved dietary choices, avoidance of risky sexual behaviour, violence and injury prevention. One initiative that could help to dissipate social networking use for means of chronic disease health information could be to create informative web-pages about chronic diseases within specifically designed scientific social networks, or social networks specifically aimed at people with chronic diseases.

### Electronic mailing lists

Electronic mailing lists among a community of subscribers can be used to spread information in clinical settings among providers or patients. Such mailing lists operate as channels for the diffusion of information, innovations and resources to improve care.

### Web based learning

Web-based learning is a tool to provide continuing training to increase the number and skills of health care workers and patients. This learning method allows for decentralised training without geographical and financial barriers. A potential limitation is equipment and broad-band dependence and also limitation for specialist training that requires face-to-face interaction [46]. One example of training by means of a web based platform is the “Enlace Hispanoamericano de Salud”, EHAS Project (meaning Link Latin American Health), which has helped to establish tele-medicine in rural areas of South America.

### Maps

Maps have long been crucial in the planning of disease control. An example is the eradication program in the US in the early 20th century; conducting a scholar survey in each county in the southern states [47].

New technologies and strong epidemiological support have improved the understanding where Neglected Tropical Diseases (NTD) can occur in recent years. Mobile phone technology coupled with GPS and Google maps provides electronic data entry at the point of collection and rapid transmission of information not only place but weather and water supply. Mobile application can collect mapping data and also make the maps available publically via the web [48]. An example of mapping NTD is the African Programme for Onchocerciasis Control (APOC), which is developing Rapid Epidemiological Mapping of Onchocerciasis (REMO).

### Telemedicine

Telemedicine aims to benefit patients who live in isolated communities by giving them a means to access healthcare information from doctors or specialists without having to visit them in person [49].

Together with mobile technology, healthcare professionals in multiple locations will be able to share information and discuss patient issues. Remote patient monitoring through mobile devices can reduce the need for outpatient visits and enable remote prescription verification and drug administration oversight. The downsides of telemedicine include the cost of telecommunication and data management equipment and also the technical training for medical personnel along with the patients who will be using it [50].

Both EHAS (Enlace Hispanoamericano de Salud) meaning “Link Latin-American Health” and Satellite African eHealth validation (SAHEL) in Africa are examples of initiatives related to the development of tele-medicine within rural regions.

### mHealth

mHealth is a developing and expanding market at a worldwide level. Businesses developing such technologies are increasingly expanding into the domain of healthcare at both an individual and collective level. Increasingly more and more of the population are utilising smartphones with applications capable of sharing information across social networks and other platforms used by individuals interested in health related information. Due to this, the use of smartphones to access information across the world only continues to increase and each time medicine is creating both more and increasingly advanced technological resources which can be accessed on a smartphone.

Different pilot studies have highlighted the impact of mobile technology on communicable diseases, such as, tuberculosis, human immunodeficiency virus, and malaria [51,52].

One important solution to the lack of coverage of mHealth is the penetration of more mobile networks in rural areas and the lowering costs of mobile handsets.

A recent meta-analysis published by Beratarrechea et al., showed that mHealth positively impacted on chronic disease outcomes, improving attendance rates (6 studies), clinical outcomes (6 studies), health-related-quality of life (2 studies), and cost-effective (3 studies) [40]. mHealth is a promising cost-effective tool to improve the health care assistance of elderly people in developing countries.

Two recent systematic reviews found modest and suggestive evidence for the benefits of mHealth technology (i.e. helping patients quit smoking, improving Human Immunodeficiency Virus (HIV) medication adherence, and some aspects of clinical diagnosis and management) [53,54].

An important problem to be resolved is the security issue. Education in security systems is fundamental to develop secure systems. Security in mHealth needs to define who in particular has access to a health record and ensuring that only secure, approved devices and applications can access health information. Specific rules, regulations and laws are currently pending regarding the protection of patient privacy and security.

Finally, it is important to mention examples of mHealth within developing countries such as the Mobile Health Alliance Initiative which defends the use of health related mobile technologies across the world. Another useful platform which utilises mobile devices in developing countires is Peekvision; helping to diagnose ophthalmology conditions within rural regions. Similarly, Pharasecure and Spyroxil are applications which verify the authenticity of medications.

There are also certain mobile phone applications which could be applied to chronic health conditions: “Alerhta” monitors blood pressure, “Catchmypain” monitors chronic pain, “Endomondo” which monitors lifestyle changes, both “MySugr” and “SocialDiabetes” help patients to monitor their diabetes and “Pregunta por tu Salud” (Ask about your health) helps patients to prepare for their medical consultations. There is also an application to remind patients to take their medications; “ReceurdaMed” (Remember Medications).

## Conclusions

eHealth technologies have the potential to improve access to empowered patients. Nowadays eHealth is available in different settings of our society. Aging societies worldwide reflect an accompanying increase in demand for personalized health services.

Chronic diseases have become more prevalent in developing countries within recent years. Some problems related to health care (limited resources, lack of literacy and large geographical areas) and age related problems (physical, cognitive, and visual impairment) make access to health care difficult. Although with the global expansion and penetration of Internet and mobile devices, several millions of citizens that never had regular access to a fixed-line telephone or a computer are now able to use new technologies for communication and data transfers. Innovative applications of mobile technology to existing health care delivery and monitoring systems offer great promise for improving the quality of life. This paper has showed how invaluable modern technology can be when applied to healthcare in limited resources regions, but the accessible information also needs to be validated. Health care workers need to be up to date and aware of the information and new technologies that is out there for everyone to see.

## Abbreviations

(ICT): Information and communication technology
(mHealth): Mobile Health
(NCD): Non-communicable Diseases
(SAHEL): Satellite African eHealth validation
(EHAS): Enlace Hispanoamericano de Salud [Link Latin American Health]
(HIV): Human Immunodeficiency Virus
(NTD): Neglected Tropical Diseases

## References

[1] Della Mea V (2001). What is e-health (2): the death of telemedicine?. J Med Internet Res.

[2] Eysenbach G (2001). What is e-health?. J Med Internet Res.

[3] ITU–T technology watch report: **E-health standards and interoperability.** 2012. [Internet]. [cited 2014 Oct 31]. Available from: www.itu.int/dms_pub/itu-t/oth/23/01/T23010000170001PDFE.pdf.

[4] Bashshur RL, Shannon GW (2009). History of Telemedicine: Evolution, Context, and Transformation.

[5] Wentzer HS, Bygholm A (2013). Narratives of empowerment and compliance: studies of communication in online patient support groups. Int J Med Inf.

[6] Isabalija SR, Mbarika V, Kituyi GM (2013). A framework for sustainable implementation of E-medicine in transitioning countries. Int J Telemed Appl.

[7] Gurol-Urganci I, de Jongh T, Vodopivec-Jamsek V, Atun R, Car J (2013). Mobile phone messaging reminders for attendance at healthcare appointments. Cochrane Database Syst Rev.

[8] The World in 2014: ICT Facts and Figures [Internet]. ITU. [cited 2014 Nov 3]. Available from: http://www.itu.int/en/ITU-D/Statistics/Pages/facts/default.aspx.

[9] Gracia E, Herrero J (2009). Internet use and self-rated health among older people: a national survey. J Med Internet Res.

[10] Jung M-L, Loria K (2010). Acceptance of Swedish e-health services. J Multidiscip Healthc.

[11] Cushman M, McLean R, Hill R, Beynon-Davies P, Williams MD (2008). Older people and internet engagement. Inf Technol People.

[12] World Health Organization: **Ageing and life course. Ten facts on ageing and the life course.** [Internet]. [cited 2014 Oct 31]. Available from: www.who.int/features/factfiles/ageing/ageing_facts/en/index.html.

[13] Kummervold PE, Chronaki CE, Lausen B, Prokosch H-U, Rasmussen J, Santana S, Staniszewski A, Wangberg SC (2008). eHealth trends in Europe 2005–2007: a population-based survey. J Med Internet Res.

[14] Peek STM, Wouters EJM, van Hoof J, Luijkx KG, Boeije HR, Vrijhoef HJM (2014). Factors influencing acceptance of technology for aging in place: a systematic review. Int J Med Inf.

[15] Bujnowska-Fedak MM, Pirogowicz I (2014). Support for e-health services among elderly primary care patients. Telemed J E-Health Off J Am Telemed Assoc.

[16] Morris A, Goodman J, Brading H (2007). Internet use and non-use: views of older users. Univers Access Inf Soc.

[17] Wagner N, Hassanein K, Head M (2010). Computer use by older adults: a multi-disciplinary review. Comput Hum Behav.

[18] Madden M: **Older Adults and Internet Use: (Some of) What we Know [Internet]. Pew Research Center’s Internet & American Life Project.** [cited 2014 Nov 3]. Available from: http://www.pewinternet.org/2013/10/21/older-adults-and-internet-use-some-of-what-we-know/.

[19] Mcmellon CA, Schiffman LG (2002). Cybersenior empowerment: How some older individuals Are taking control of their lives. J Appl Gerontol.

[20] Chen Y, Persson A (2002). Internet Use among young and older adults: relation to psychological well-being. Educ Gerontol.

[21] Shapira N, Barak A, Gal I (2007). Promoting older adults’ well-being through Internet training and use. Aging Ment Health.

[22] Cline RJW, Haynes KM (2001). Consumer health information seeking on the Internet: the state of the art. Health Educ Res.

[23] Jensen JD, King AJ, Davis LA, Guntzviller LM (2010). Utilization of internet technology by low-income adults: the role of health literacy, health numeracy, and computer assistance. J Aging Health.

[24] Neter E, Brainin E (2012). eHealth literacy: extending the digital divide to the realm of health information. J Med Internet Res.

[25] Carpenter BD, Buday S (2007). Computer use among older adults in a naturally occurring retirement community. Comput Hum Behav.

[26] Choi NG, Dinitto DM (2013). Internet use among older adults: association with health needs, psychological capital, and social capital. J Med Internet Res.

[27] Betjeman TJ, Soghoian SE, Foran MP (2013). mHealth in Sub-Saharan Africa. Int J Telemed Appl.

[28] Catalani C, Philbrick W, Fraser H, Mechael P, Israelski DM (2013). MHealth for HIV treatment & prevention: a systematic review of the literature. Open AIDS J.

[29] Car J, Gurol-Urganci I, de Jongh T, Vodopivec-Jamsek V, Atun R (2012). Mobile phone messaging reminders for attendance at healthcare appointments. Cochrane Database Syst Rev.

[30] Guy R, Hocking J, Wand H, Stott S, Ali H, Kaldor J (2012). How effective are short message service reminders at increasing clinic attendance? A meta-analysis and systematic review. Health Serv Res.

[31] Collins F (2012). How to fulfill the true promise of “mHealth”: Mobile devices have the potential to become powerful medical tools. Sci Am.

[32] Hewapathirana R. mHealth: Evidence and Best Practices. Sri Lanka J Bio-Med Inform [Internet]. 2010 Jul 13 [cited 2014 Nov 3];1(3). Available from: http://www.sljol.info/index.php/SLJBMI/article/view/2049.

[33] Nahm E-S, Preece J, Resnick B, Mills ME (2004). Usability of health Web sites for older adults: a preliminary study. Comput Inform Nurs CIN.

[34] Williams MV, Davis T, Parker RM, Weiss BD (2002). The role of health literacy in patient-physician communication. Fam Med.

[35] Detlefsen EG. Where Am I to Go? Use of the Internet for Consumer Health Information by Two Vulnerable Communities. 2004 [cited 2014 Nov 3]; Available from: https://www.ideals.illinois.edu/handle/2142/1733.

[36] Alpay LL, Toussaint PJ, Ezendam NPM, Rövekamp TAJM, Graafmans WC, Westendorp RGJ (2004). Easing Internet access of health information for elderly users. Health Informatics J.

[37] Chu A, Huber J, Mastel-Smith B, Cesario S (2009). “Partnering with seniors for better health”: computer use and internet health information retrieval among older adults in a low socioeconomic community. J Med Libr Assoc JMLA.

[38] Baker L, Wagner TH, Singer S, Bundorf MK (2003). Use of the Internet and e-mail for health care information: results from a national survey. JAMA.

[39] WHO | Global status report on noncommunicable diseases 2010 [Internet]. WHO. [cited 2014 Nov 3]. Available from: http://www.who.int/nmh/publications/ncd_report2010/en/.

[40] Beratarrechea A, Lee AG, Willner JM, Jahangir E, Ciapponi A, Rubinstein A (2014). The impact of mobile health interventions on chronic disease outcomes in developing countries: a systematic review. Telemed J E-Health Off J Am Telemed Assoc.

[41] WHO | Global Observatory for eHealth series - Volume 3 [Internet]. WHO. [cited 2014 Nov 3]. Available from: http://www.who.int/goe/publications/ehealth_series_vol3/en/.

[42] Collins LM, Baker TB, Mermelstein RJ, Piper ME, Jorenby DE, Smith SS, Christiansen BA, Schlam TR, Cook JW, Fiore MC (2011). The multiphase optimization strategy for engineering effective tobacco use interventions. Ann Behav Med Publ Soc Behav Med.

[43] Tomlinson M, Rotheram-Borus MJ, Swartz L, Tsai AC (2013). Scaling up mHealth: where is the evidence?. PLoS Med.

[44] Boutayeb A (2006). The double burden of communicable and non-communicable diseases in developing countries. Trans R Soc Trop Med Hyg.

[45] Arah OA, Ogbu UC, Okeke CE (2008). Too poor to leave, too rich to stay: developmental and global health correlates of physician migration to the United States, Canada, Australia, and the United Kingdom. Am J Public Health.

[46] Kahn JG, Yang JS, Kahn JS (2010). “Mobile” health needs and opportunities in developing countries. Health Aff Proj Hope.

[47] Humphreys M (2009). How four once common diseases were eliminated from the American South. Health Aff Proj Hope.

[48] Smith JL, Haddad D, Polack S, Harding-Esch EM, Hooper PJ, Mabey DC, Solomon AW, Brooker S (2011). Mapping the global distribution of trachoma: why an updated atlas is needed. PLoS Negl Trop Dis.

[49] Berman M, Fenaughty A (2005). Technology and managed care: patient benefits of telemedicine in a rural health care network. Health Econ.

[50] Hjelm NM (2005). Benefits and drawbacks of telemedicine. J Telemed Telecare.

[51] Mbuagbaw L, van der Kop ML, Lester RT, Thirumurthy H, Pop-Eleches C, Ye C, Smieja M, Dolovich L, Mills EJ, Thabane L (2013). Mobile phone text messages for improving adherence to antiretroviral therapy (ART): an individual patient data meta-analysis of randomised trials. BMJ Open.

[52] Crankshaw T, Corless IB, Giddy J, Nicholas PK, Eichbaum Q, Butler LM (2010). Exploring the patterns of use and the feasibility of using cellular phones for clinic appointment reminders and adherence messages in an antiretroviral treatment clinic, Durban, South Africa. AIDS Patient Care STDs.

[53] Free C, Phillips G, Watson L, Galli L, Felix L, Edwards P, Patel V, Haines A (2013). The effectiveness of mobile-health technologies to improve health care service delivery processes: a systematic review and meta-analysis. PLoS Med.

[54] Free C, Phillips G, Galli L, Watson L, Felix L, Edwards P, Patel V, Haines A (2013). The effectiveness of mobile-health technology-based health behaviour change or disease management interventions for health care consumers: a systematic review. PLoS Med.

